# Does C1 esterase inhibitor play a role in post COVID-19 neurological symptoms? A randomized, double-blind, placebo-controlled, crossover, proof-of-concept study

**DOI:** 10.3389/fneur.2025.1523814

**Published:** 2025-11-06

**Authors:** Isaac Melamed, Caley Buckley, Mary Ellen Bayko, Joe Lynn Williams, Noga Or-Geva

**Affiliations:** 1 IMMUNOe Research Center, Centennial, CO, United States; 2 Department of Neurology and Neurological Sciences, Stanford University, Stanford, CA, United States

**Keywords:** C1-INH, cognitive dysfunction, complement activation, fatigue, immune system, neurological symptoms, SARS-CoV-2

## Abstract

**Background:**

Many patients with severe acute respiratory syndrome coronavirus 2 (SARS-CoV-2) infection experience neurologic changes post-infection, which has been hypothesized to be due to dysregulation in the infectious-immune axis that leads to a neuro-immune response. This immune dysfunction has been termed “Alzheimer’s of the Immune System” or AIS and there are several immune factors that may play a key role. These include, among others, complement activation due to low levels of C1-esterase inhibitor (C1-INH) and function, and a decrease in signaling of Toll-like receptor (TLR)-3. We propose that C1-INH replacement may upregulate the immune dysfunction, thereby improving neurological symptoms.

**Methods:**

In this randomized, double-blind, placebo-controlled, crossover, proof-of-concept study, adults experiencing SARS-CoV-2 post-viral fatigue syndrome for >4 weeks post-recovery from coronavirus disease 2019 (COVID-19) infection were randomized 1:1 to two arms: Arm 1 (C1-INH for 8 weeks, then placebo for 8 weeks) or to Arm 2 (placebo for 8 weeks, then C1-INH for 8 weeks). Patients were assessed for adult executive function, abnormal cognitive decline, depression [Beck Depression Inventory-II (BDI-II)], migraine, fatigue [Fatigue Severity Scale (FSS)] and pain (Short-form McGill Pain Questionnaire). Percent change in TLR signaling in response to zymosan was compared with controls at baseline, Week 8 and Week 16. Safety was assessed throughout.

**Results:**

At this interim analysis, 36 patients with SARS-CoV-2 post-viral fatigue syndrome had completed the two 8-week treatment periods. In Arm 1, trends toward improvements from baseline at Week 8 of C1-INH therapy were observed in BDI-II score (−8.7 points), mean FSS score (0.6 points), and mean McGill Pain Questionnaire score (−0.4 points). These improvements were either sustained or worsened at Week 16, following crossover to placebo. The outcomes in Arm 2 were compatible with those in Arm 1. Patients with SARS-CoV-2 post-viral fatigue syndrome had low levels of TLR-related signaling biomarkers compared with healthy controls.

**Conclusion:**

This proof-of-concept study demonstrates sustained dysregulation of the immune system after COVID-19 infection. Improvements in depression, fatigue, and pain were observed with C1-INH treatment in patients with SARS-CoV-2 post-viral fatigue syndrome, indicating C1-INH may be a potential therapeutic target.

**Clinical trial registration:**

## 1 Introduction

Findings suggest that neurological symptoms (memory issues, cognitive changes, tremors, etc.) after an infection may be related to a form of post-infectious autoimmunity. Various disorders are associated with neurological and cognitive changes, which may occur post-infection, including chronic fatigue syndrome (1), pediatric acute-onset neuropsychiatric syndrome (PANS) (2), Lyme disease (3, 4), and autism (5). Notably, approximately 30–80% of patients with persistent coronavirus disease 2019 (COVID-19) symptoms (long COVID) develop fatigue and cognitive deficits lasting 1–6 months, including reduced executive functions, memory, processing speed, and attention (6–9).

The key mechanism for brain autoimmunity may be dysfunctional neuroimmune responses to various infectious pathogens. In 2016, we coined the term “Alzheimer’s of the Immune System” (AIS) to identify this syndrome (10). In certain patients, a memory defect of the immune system may result in failure to recognize infectious pathogens that cause the neurological diseases (10). This memory defect may create a neurological storm that likely includes various factors, including low levels of C1-esterase inhibitor (C1-INH) resulting in complement activation, reduction in Toll-like receptor (TLR)-3 signaling, and low response to T-cell antigens (10, 11).

The complement system may be crucial in AIS. As well as directly interacting with pathogens, the complement system forms a bridge between the innate and adaptive immune responses (12–15). For the adaptive immune response, complement components are involved in the regulation of T-cell and B-cell activation (14). For the innate immune response, complement engages in signaling crosstalk with TLRs to coordinate immune and inflammatory responses. Complement component C1 triggers the classical pathway for complement activation; as such, C1-INH plays an important role as a check against uncontrolled complement activation (13, 16). We hypothesize that the dysregulation of the complement and TLR signaling pathways may lead to a dampening of the response to infection and, therefore, persistent post-infectious neuroinflammation.

Dysregulation of the complement system has been linked with various neurodevelopmental disorders, including schizophrenia, autism spectrum disorder, anxiety and mood disorders (17). Several non-clinical studies suggest that targeting this system through treatment with C1-INH can improve neurological function, potentially through anti-inflammatory effects (18–21). In order to further understand the immune mechanisms that lead to post-infectious neuroinflammation, we report an ongoing study investigating post-viral fatigue in patients experiencing long COVID. We investigated whether recombinant human C1-INH (RUCONEST^®^, Pharming Group N. V.) therapy may upregulate the immune dysfunction and therefore improve neurological symptoms, compared with placebo.

## 2 Materials and methods

### 2.1 Study design and oversight

This ongoing Phase 1, randomized, double-blind, placebo-controlled, crossover, proof-of-concept study (ClinicalTrials.gov number, NCT04705831) comprises a 2-week screening period, 8-week initial treatment period, and 8-week crossover treatment period. Study visits occurred during screening (Weeks −2 or −1; when baseline assessments were conducted) and once per week in both treatment periods.

Patients were randomized 1:1 to two arms. C1-INH or placebo were administered once a week, from the first day of Week 0. In Arm 1, patients were treated initially with C1-INH (last dose at Week 7) followed by crossover to placebo (last dose at Week 15), and in Arm 2 with placebo (last dose at Week 7) followed by crossover to C1-INH (last dose at Week 15). Each dose of C1-INH (4,200 U once a week) and placebo was administered intravenously for approximately 5 min.

Randomization was conducted by pharmacy staff using a 10-block method. Pharmacy staff were not blinded and maintained drug accountability records. All other study staff were blinded, including investigators. Should an adverse drug reaction or serious adverse event (SAE) occur, investigators could request unblinding. In the event of unblinding, study participation would cease.

C1-INH (RUCONEST^®^, Pharming Group N. V.) was supplied in single-use 25 mL glass vials, each containing 2,100 U C1-INH lyophilized powder for reconstitution in 14 mL of sterile water. The reconstituted solution contained 150 IU/mL C1-INH and was clear and colorless. Placebo was sterile saline, administered at the same volume as the study medication, using the same pumps and infusion rates.

A local ethics committee provided unconditional written approval for the study. The study was conducted according to local regulatory requirements and International Conference for Harmonisation Good Clinical Practice guidelines.

### 2.2 Patients

Patient eligibility criteria are shown in Supplementary Table 1. In brief, eligible patients were adults ≥18 years of age experiencing severe acute respiratory syndrome coronavirus 2 (SARS-CoV-2) post-viral fatigue syndrome for more than 4 weeks after recovering from COVID-19 infection, documented by polymerase chain reaction (PCR) or spike antibody testing, who provided written informed consent before any study procedures were conducted. There were no cases of severe COVID or hospitalizations in the patient population studied.

### 2.3 Endpoints

Neuropsychological outcomes were assessed using the following scales: Beck Depression Inventory-II (BDI-II), Behavior Rating Inventory of Executive Function-Adult (BRIEF-A), Repeatable Battery for the Assessment of Neuropsychological Status (RBANS), and Montreal Cognitive Assessment (MoCA). BDI-II scores indicate no depression (0–9) or depression that is mild–moderate (10–18), moderate–severe (19–29), and severe (30–63) (22). BRIEF-A captures executive functions across two domains [Behavioral Regulation Index (BRI) and Metacognition Index (MI)], resulting in the Global Executive Composite (GEC) score (23). Lower values represent less impairment. RBANS captures cognitive function (immediate memory, visuospatial/constructional, language, attention, and delayed memory), with scores ≥70, 55–69, and <54 indicating mild, moderate, and severe impairment, respectively (24). MoCA also assesses cognitive function, with a normal score considered to be ≥27.4 (25).

Patient-reported pain, fatigue, and migraine outcomes were assessed using the following questionnaires: Short-form (SF) McGill Pain Questionnaire, Fatigue Severity Scale (FSS), Migraine Disability Assessment (MIDAS), and six-item Headache Impact Scale (HIT-6). The SF McGill Pain scoring scale ranges from 0 (no pain) to 10 (worst pain imaginable) (26). FSS scoring ranges from 1 to 7, with higher scores indicating worse fatigue (27). MIDAS measures both the number of days in the last 3 months that the patient had a headache and uses a scoring scale for pain, ranging from 0 (no pain) to 10 (pain as bad as it can be) (28). HIT-6 (score range, 36–78) was designed as an instrument to measure the impact headaches have on the ability to function at work, in school, and in social situations, with reductions showing improvement (29).

TLR activity was evaluated by measuring three inflammatory markers [tumor necrosis factor alpha (TNFα), interleukin (IL)-1β, and IL-6] in patients before treatment and comparing these to healthy controls. Blood (10 mL) was collected and analyzed by ARUP laboratories. TLR signaling was tested independently by stimulation with TLR6-TLR2 ligand using zymosan cell wall particles from *Saccharomyces cerevisiae* in a peripheral blood mononuclear cell (PBMC) culture. PBMC production of TNFα, IL-1β, and IL-6 was determined by multiplex bead assay.

Efficacy and safety outcomes were assessed in the initial treatment period (up to Week 8) and crossover treatment period (up to Week 16).

### 2.4 Statistical analysis

This ongoing, exploratory, proof-of-concept study used descriptive statistics. No sample size calculation was conducted. Analysis was performed for participants with available data. For responder analyses, patients with any improvement from baseline were classified as responders and patients with missing values were classified as non-responders. Statistical analyses were performed in GraphPad Prism (10.2.3). Shapiro–Wilk normality tests were performed prior to the Mann–Whitney U Test for TLR activity between patients and healthy controls, and the Wilcoxon matched-pairs signed rank test for depression, fatigue, and pain scores (between baseline and Week 8, and baseline and Week 16). *p* < 0.05 were considered statistically significant.

## 3 Results

### 3.1 Baseline characteristics and patient disposition

**Figure 1:**
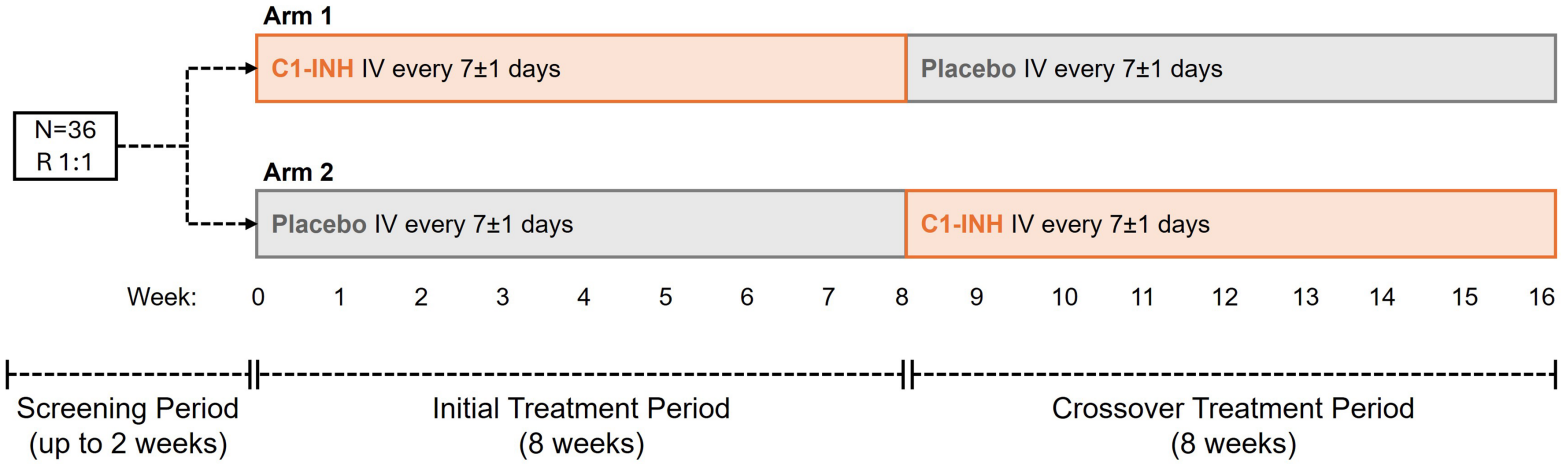
Study design. Thirty-six patients with SARS-CoV-2 post-viral fatigue syndrome were randomized 1:1 to Arms 1 and 2. In Arm 1, patients received C1-INH from Week 0, crossing over to placebo from Week 8. In Arm 2, patients received placebo from Week 0, crossing over to C1-INH from Week 8. C1-INH was dosed at 4,200 U once a week. C1-INH, C1 esterase inhibitor; IV, intravenous; R, randomized.

This ongoing study commenced in December 2020, during which time the predominant strain of SARS-CoV-2 was the Alpha variant (B.1.1.7). The time from infection to enrollment was between 4 weeks and 3 months for all patients and was not related to the acute phase of COVID infection. Overall, 36 participants with SARS-CoV-2 post-viral fatigue syndrome were randomized 1:1 to Arms 1 and 2 (Figure 1). All 36 patients completed initial 8-week and crossover 8-week treatment periods.

At baseline, patient demographics were comparable between the treatment arms (Table 1). Nineteen patients were vaccinated against SARS-CoV-2 at study enrollment. Preexisting conditions included attention deficit hyperactivity disorder (ADHD; *n* = 7), post-traumatic stress disorder (*n* = 1), and documented insomnia (*n* = 3). At enrollment, 12 patients began taking antidepressive agents and 15 began taking anti-anxiety medication post-COVID-19 infection. Four patients had been taking sleep medication prior to infection, and two were treated for atypical seizure after infection. Mean age was 48.7 years (standard deviation 11.9 years). Most patients were female (80.5%) and White non-Hispanic (91.7%).

**Table 1:** Baseline demographics and neuropsychological measures.

Characteristic*	Arm 1: C1-INH → PBO^†^ (*N* = 18)	Arm 2: PBO → C1-INH^†^ (*N* = 18)
Age, n		
Mean	49.6 (12.5)	47.8 (11.3)
Median (range)	49 (26–75)	45 (28–70)
Female, n (%)	14 (77.8)	15 (83.3)
Race/ethnicity, n (%)		
White non-Hispanic	16 (88.9)	17 (94.4)
Asian	2 (11.1)	1 (5.6)
BDI-II score	*n* = 18 27.2 (15.9)	*n* = 18 19.7 (9.3)
FSS score	*n* = 14 2.71 (2.40)	*n* = 15 3.53 (1.89)
SF McGill pain score	*n* = 16 3.38 (2.78)	*n* = 13 2.92 (2.16)
BRIEF-A GEC T-score	*n* = 18 66.0 (11.8)	*n* = 18 66.1 (14.1)
BRIEF-A BRI T-score	*n* = 18 57.3 (9.1)	*n* = 18 62.4 (13.6)
BRIEF-A MI T-score	*n* = 18 71.6 (13.5)	*n* = 18 66.9 (14.6)
RBANS score	*n* = 18 97.1 (16.3)	*n* = 18 97.3 (16.1)
MoCA total score	*n* = 18 26.0 (2.5)	*n* = 18 26.3 (2.0)
MIDAS quantity score	*n* = 18 25.9 (27.5)	*n* = 16 21.5 (23.0)
MIDAS pain severity score	*n* = 18 5.41 (2.37)	*n* = 16 4.06 (2.36)
HIT-6 score	*n* = 18 56.6 (10.8)	*n* = 17 54.1 (11.6)

*Mean (SD), unless stated otherwise. †Patients were treated once a week, in Arm 1 with C1-INH (last dose at Week 7) and crossover to placebo (last dose at Week 15), and in Arm 2 with placebo (last dose at Week 7) and crossover to C1-INH (last dose at Week 15). BDI-II, Beck Depression Inventory-II; BRIEF-A, Behavior Rating Inventory of Executive Function-Adult; BRI, Behavioral Regulation Index; FSS, Fatigue Severity Scale; GEC, Global Executive Composite; HIT, Headache Impact Scale; MI, Metacognition Index; MIDAS, Migraine Disability Assessment; MoCA, Montreal Cognitive Assessment; RBANS, Repeatable Battery for the Assessment of Neuropsychological Status; SF McGill pain, Short Form McGill Pain Questionnaire.

Neuropsychological measures were comparable in Arms 1 and 2 at baseline, with some imbalances. In Arms 1 and 2, mean BDI-II scores were 27.2 and 19.7 (based on a 0–63 point scale), mean FSS scores were 2.7 and 3.5 (based on a 1–7 point scale), and SF McGill Pain scores were 3.4 and 2.9 (based on a 0–10-point scale), respectively (Table 1). Both mean BDI-II scores indicated moderate–severe depression.

### 3.2 Immunological biomarkers

**Figure 2:**
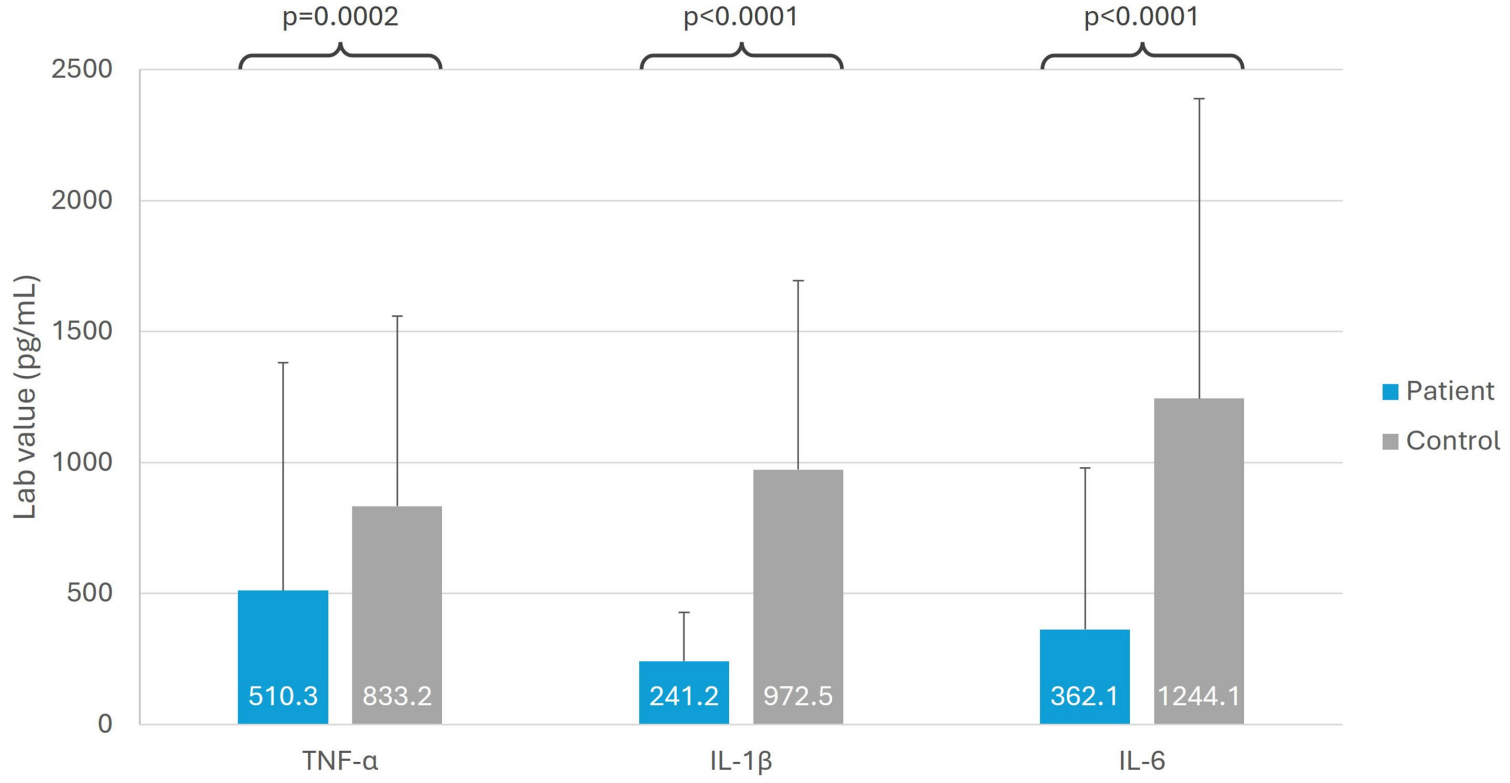
Immunological biomarkers in patients with SARS-CoV-2 post-viral fatigue syndrome versus healthy controls. IL, interleukin; TNF-α, tumor necrosis factor alpha.

When assessed at baseline, patients with SARS-CoV-2 post-viral fatigue syndrome (*n* = 36) had significantly lower mean levels of TLR-related signaling biomarkers, compared with healthy controls (*n* = 36) (Figure 2). Patients with SARS-CoV-2 had a 37.7% reduction in TNF-*α* signaling (*p* = 0.0002), 75.0% reduction in IL-1β signaling (*p* < 0.0001), and 70.0% reduction in IL-6 signaling (*p* < 0.0001) as compared with healthy controls.

### 3.3 Efficacy outcomes

#### 3.3.1 Cognitive changes

**Figure 3:**
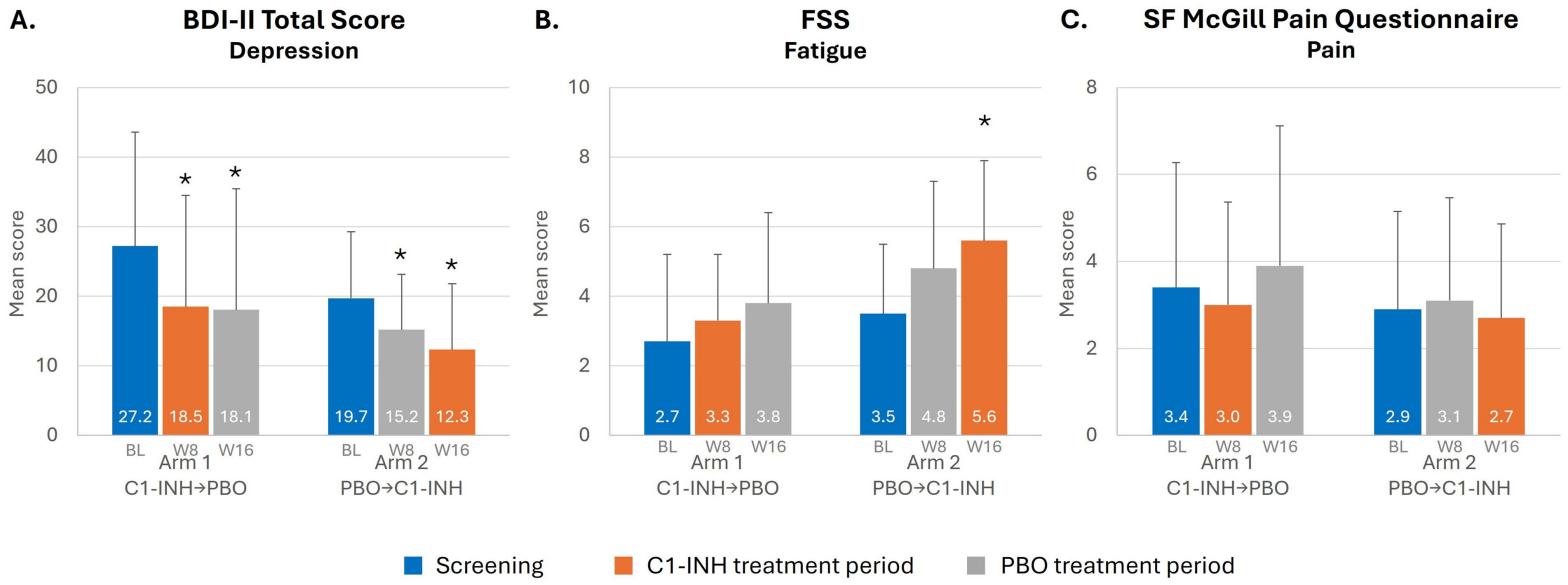
Depression, pain and fatigue in patients with SARS-CoV-2 post-viral fatigue syndrome. *Indicates *p* < 0.05. BL, baseline (value during the screening period); BDI-II, Beck Depression Inventory-II; C1-INH, C1-esterase inhibitor; FSS, Fatigue Severity Scale; PBO, placebo; SF, short-form; W, week.

Trends toward improvements were observed in depressed patients based on BDI-II score during treatment with C1-INH (Figure 3A). In Arm 1, mean BDI-II score improved at Week 8 during treatment with C1-INH [decreasing by 32.0% (8.7 points) from baseline; *p* = 0.0010] and was maintained after crossover to placebo (*p* = 0.0003). In Arm 2, mean BDI-II score improved slightly at Week 8 with placebo [decreasing by 22.8% (4.5 points) from baseline; *p* = 0.0132] and, notably, improved further at Week 16 during treatment with C1-INH [by 19.1% (7.4 points) from baseline; *p* = 0.0036].

No notable improvements with C1-INH treatment were observed in other rating scales, including in executive function (BRIEF-A) and cognitive function (RBANS and MoCA) (Supplementary Table 2). For Arm 1 starting on C1-INH treatment, the mean percent change in RBANS showed an overall improvement in cognition with an increase of 1.9% and a reduction of 4.6% after moving to placebo. In Arm 2, mean RBANS score increased by 2.6% while on placebo followed by a slight decrease of 0.4% while on C1-INH.

**Figure 4:**
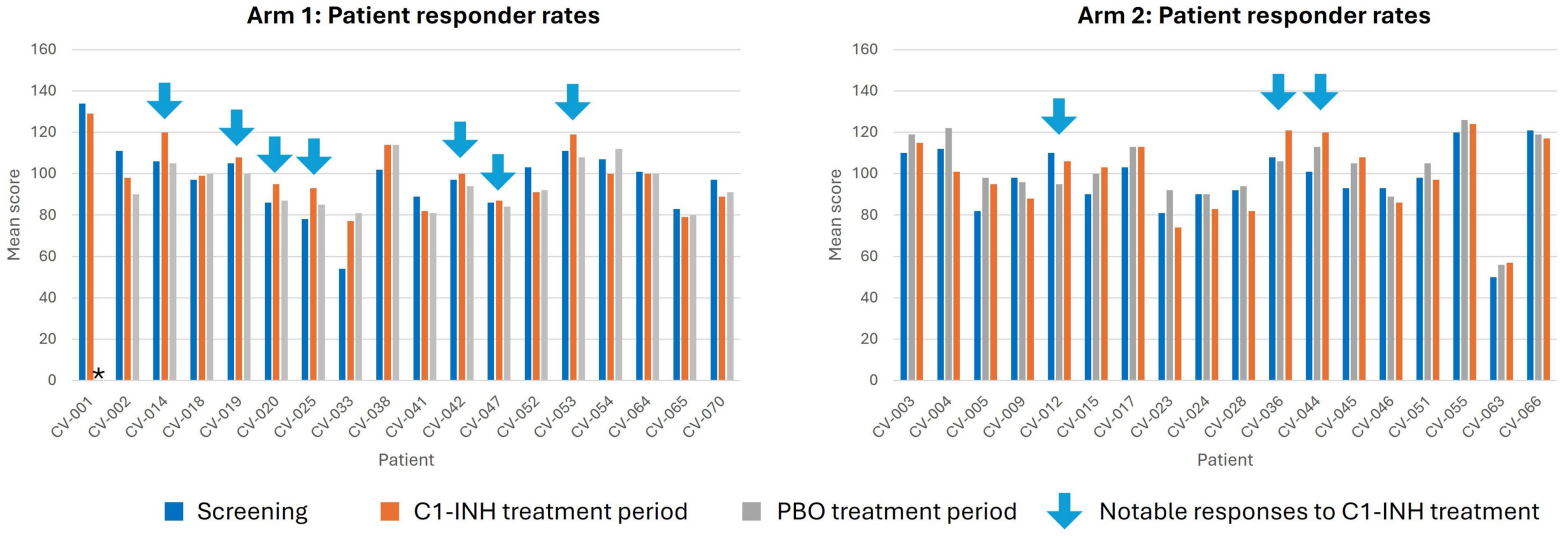
Responder analysis for RBANS score at baseline, Week 8, and Week 16. *Indicates measure not reported. C1-INH, C1-esterase inhibitor; CV, clinical volunteer; PBO, placebo.

In an analysis of RBANS score by patient, seven patients in Arm 1 and three patients in Arm 2 were observed to have improved RBANS score after C1-INH treatment (Figure 4). In general, patients with no other underlying neurological symptoms before SARS-CoV-2 infection had better RBANS responses, and those with conditions such as ADHD and depression had a worse response.

#### 3.3.2 Fatigue, migraine, and headache

Mean FSS score improved at Week 8 during treatment with C1-INH (increasing by 0.6 points from baseline; *p* = 0.5337) in Arm 1, with continued improvement at Week 16 following crossover to placebo (increased by 1.1 points from baseline; *p* = 0.5137) (Figure 3B). In Arm 2, a similar trend was observed, with FSS score increasing by 1.3 points from baseline at Week 8 with placebo (*p* = 0.0664), and then further improvement after crossover to C1-INH treatment, increasing by 2.1 points from baseline at Week 16 (*p* = 0.0078).

Outcomes assessing headache (HIT-6) or migraine (MIDAS) did not observe any notable improvements with C1-INH treatment in Arm 1 or Arm 2 (Supplementary Table 2).

##### 3.3.3 Pain

Mean SF McGill Pain score in Arm 1 improved at Week 8 during treatment with C1-INH (decreasing by 0.4 points from baseline; *p* = 0.6270) and then worsened at Week 16 following crossover to placebo (increasing by 0.5 points from baseline; *p* = 0.2031) (Figure 3C). In Arm 2, SF McGill Pain score increased slightly at Week 8 of placebo (by 0.2 points from baseline; *p* = 0.3750) and decreased slightly at Week 16 after crossover to C1-INH (by 0.2 points from baseline; *p* = 0.1719).

### 3.4 Safety

No new safety signals were identified (Table 2). No SAEs were observed. Most adverse events were mild in intensity in each treatment group. One SAE (fatigue) was observed in the Arm 2 placebo group.

**Table 2:** Summary of adverse events.

Number of adverse events	Arm 1 (*N* = 18)		Arm 2 (*N* = 18)	
	C1-INH*	Placebo*	Placebo*	C1-INH*
Any adverse event	93	30	118	74
Intensity				
Mild	88	29	93	64
Moderate	4	1	19	8
Severe	0	0	1	0
Unknown	1	0	5	2
Treatment causality				
Not related	55	23	43	38
Possibly related	30	7	59	34
Probably related	7	0	12	2
Unknown	1	0	4	0
≥2 adverse events reported for either treatment in either arm				
Anxiety/panic	0	0	3	0
Appetite loss	4	0	5	0
Brain fog	3	0	5	1
Burning in nose	2	0	0	0
Burning/watery eyes	2	0	0	0
Body/back ache	3	0	4	1
Constipation	2	0	0	0
Dehydration/dry mouth/thirst	1	0	2	0
Diarrhea/loose stool	4	1	1	6^†^
Dizziness	3	2	2	7
Elevated blood pressure	2	0	1	2
Elevated creatinine	0	0	3	0
Fatigue/lethargy	13	1	12	4
Fever/flu or cold symptoms	0	2	1	0
Headache/migraine	14	6	15	7
Head pressure	0	0	2	0
Infection^‡^	2	1	3	2
Insomnia/sleeping difficulty	1	0	6	1
Irritability	2	0	0	0
Malaise	0	0	5	0
Nausea	3	2	12	6
Neck pain	0	0	0	2
Rash	2	3	0	0
Sore throat	0	1	0	3
Stomach-ache	2	1	1	1
Vertigo	2	0	1	0
Vomiting	0	1	0	2
Worsening of depression	2	0	0	0

*Patients were treated once a week, in Arm 1 with C1-INH (last dose at Week 7) and crossover to placebo (last dose at Week 15), and in Arm 2 with placebo (last dose at Week 7) and crossover to C1-INH (last dose at Week 15). †Another patient in Arm 2, treated with C1-INH, also reported an unspecified digestive issue. ‡Infections were: COVID-19 (*n* = 1) and urinary tract (*n* = 1) in Arm 1, C1-INH therapy. Urinary tract (*n* = 1) in Arm 1, placebo. Viral (*n* = 1), respiratory (*n* = 1), and urinary tract (*n* = 1) in Arm 2, placebo. Respiratory (*n* = 1) and yeast (*n* = 1) in Arm 2, C1-INH therapy.

## 4 Discussion

In this ongoing proof-of-concept study, we observed trends toward improvement in depression, fatigue, and pain during 8 weeks of C1-INH therapy in patients with SARS-CoV-2 post-viral fatigue syndrome. Furthermore, we demonstrated reduced TLR signaling components upon stimulation in patients with SARS-CoV-2 post-viral fatigue syndrome, in comparison with healthy controls. Although not statistically significant, we found that patients with no underlying neurological symptoms such as ADHD or depression did not respond as well as those with no underlying conditions.

In this study, a dysfunction in TLR signaling response (TNFα, IL-1β, and IL-6) was demonstrated in patients with SARS-CoV-2 post-viral fatigue syndrome, suggesting a possible dysregulation of innate immunity in these patients. Dysregulation of the innate immune system has been reported in patients with SARS-CoV-2 post-viral fatigue syndrome in several other studies (30–32) and are compatible with our previous reports of reduced TLR-3 expression following other infections (10). Innate immune cells have been shown to play a key role in neuropathic pain, being the first line of immunosurveillance and activation of neurogenic inflammation (33). Inflammatory processes, involving TLR-related molecules, have also previously been implicated in their pathogenesis of depression and fatigue (34–36). The dysfunction observed in TLR signaling in patients with SARS-CoV-2 post-viral fatigue syndrome may therefore play a role in the neurological symptoms of pain, depression and fatigue.

Complement has been previously shown to engage in signaling crosstalk with the TLR and acts as a bridge between the innate and adaptive immune responses to coordinate immune responses (12–15). Therefore, it is notable we observed not only dysfunction in the innate immune response but trends toward improvements in depression, fatigue, and pain during treatment with C1-INH. C1-INH plays a major role in controlling complement activation and has been previously reported to improve neurological functions by exerting an anti-inflammatory effect (18, 19). Complement may therefore play a contributory role in the persistent post-infection inflammation we observed through crosstalk between the innate and adaptive immune systems.

Dysregulation of the immune system and neurological changes have been described previously following other infections, such as Epstein Barr virus in MS (10), PANS (2), Lyme disease (37) and myalgic encephalomyelitis/chronic fatigue syndrome (ME/CFS) (38) among others (10). Lyme disease, caused by Borrelia bacteria, is associated with various neurological symptoms such as headache, fatigue, myalgia and arthralgia, with emerging evidence that attributes clinical manifestation to abnormalities in the host immune response (37). Further, SARS-CoV-2 post-viral fatigue syndrome shares similar symptoms to ME/CFS, another chronic condition characterized by neurological symptoms that often occurs following an “infectious-like” illness (38). Like SARS-CoV-2 post-viral fatigue syndrome and Lyme disease, immunological and metabolic abnormalities have also been described in ME/CFS (38) underlying the hypothesis that immune dysregulation may alter the relationship of infectious immune and lead to the neuro-immune response observed.

This proof-of-concept study has notable limitations and strengths. This exploratory analysis was limited by sample size, with some imbalance observed at baseline in disease characteristics and medication use that may have influenced treatment outcomes. Most patients at baseline had very mild RBANS and MoCA scores, indicating no cognitive impairment and, therefore, a possible ceiling effect may explain the lack of improvement in RBANS and MoCA. As some patients had neurological symptoms before they had COVID-19, which was not factored into the randomization, this could also confound the interpretation of the results. However, we hypothesize that some of the population with pre-existing neurological conditions may not have had post-SARS-CoV-2 fatigue syndrome, therefore affecting treatment outcomes with C1-INH. This is further supported through the responder analysis of RBANS score, in which improved cognitive was noted in many patients. Most patients were White non-Hispanic, thus limiting generalizability. Additionally, although the central hypothesis at the heart of this study involves both innate and adaptive immunity, linked through complement system crosstalk, the absence of direct complement activation markers and adaptive immunity data limits our ability to confirm these mechanistic pathways. As a result, further studies are warranted to confirm this. Regarding strengths, the study benefitted from a randomized, double-blind, crossover design, with patients serving as their own placebo controls, theoretically reducing some confounders and variability.

This study provides further evidence to support our hypothesis of AIS, by aiding our understanding of the role of the innate and adaptive immune response in SARS-CoV-2 post-viral fatigue syndrome. Furthering the understanding of the infectious-immune axis is important to provide the tools to identify treatment and management of neurologic changes that occur after infection. Future work will seek to better comprehend the role of C1-INH across other AIS conditions, and to explore further the role of the innate immune response and TLR signaling in SARS-CoV-2 post-viral fatigue syndrome. An open-label, Phase 2 study has been planned to further evaluate the role of C1-INH in patients with SARS-CoV-2 post-viral fatigue syndrome.

In conclusion, this proof-of-concept study demonstrates sustained dysregulation of the immune system in patients with SARS-CoV-2 post-viral fatigue syndrome and that treatment with C1-INH can improve associated symptoms of depression, fatigue, and pain. The results suggest that the complement system may play a key contributory role in this immune deficiency and could be a potential therapeutic target in patients with SARS-CoV-2 post-viral fatigue syndrome, though further studies are needed to confirm this.

## Data Availability

The original contributions presented in the study are included in the article/Supplementary material, further inquiries can be directed to the corresponding author.

## Glossary

AIS: Alzheimer’s of the Immune System
BDI-II: Beck Depression Inventory-II
BRI: Behavioral Regulation Index
BRIEF-A: Behavior Rating Inventory of Executive Function-Adult
C1-INH: C1-esterase inhibitor
COVID-19: coronavirus disease 2019
FSS: Fatigue Severity Scale
GEC: Global Executive Composite
HIT-6: six-item Headache Impact Scale
IL: interleukin
ME/CFS: myalgic encephalomyelitis/chronic fatigue syndrome
MI: Metacognition Index
MIDAS: Migraine Disability Assessment
MoCA: Montreal Cognitive Assessment
PANS: pediatric acute-onset neuropsychiatric syndrome
PBMC: peripheral blood mononuclear cell
RBANS: Repeatable Battery for the Assessment of Neuropsychological Status
SAE: serious adverse event
SARS-CoV-2: severe acute respiratory syndrome coronavirus 2
SF: Short-form
TLR: Toll-like receptor
TNFα: tumor necrosis factor alpha
