# Supplementary material for: Does C1 esterase inhibitor play a role in post COVID-19 neurological symptoms? A randomized, double-blind, placebo-controlled, crossover, proof-of-concept study
Source: Front Neurol. 2025 Nov 6;16:1523814. doi: 10.3389/fneur.2025.1523814 (PMC12631290; doi:10.3389/fneur.2025.1523814)
Supplement: Supplementary file 1 [file Table_1.docx]

Supplementary Material

# Supplementary Figures and Tables

## Supplementary Tables

**Supplementary Table 1.** Patient eligibility criteria

| **Inclusion criteria** |
| --- |
| 1. Age 18 and older, male or female. 2. Previous confirmed diagnosis of SARS-CoV-2 Experiencing SARS-CoV-2 post-viral fatigue syndrome 4 weeks after recovery from SARS-CoV-2 infection. 3. Experiencing neurological symptoms including fatigue. 4. Willing to comply with all aspects of the protocol, including blood draws. 5. Able to understand and fully participate in study activities and to provide informed consent. 6. Female patients, if sexually active and of childbearing potential, had to be willing to use an acceptable form of contraception. Acceptable forms of contraception were defined as those with a failure rate of <1% when properly applied and include: a combination oral pill, some intra-uterine devices, and a sterilized partner in a stable relationship. Female patients could not be pregnant, not planning to become pregnant, and not be actively breastfeeding throughout the study. |
| **Exclusion criteria** |
| 1. Receiving any form of C1-INH therapy (acute or prophylactic treatment). 2. History or suspicion of allergy to rabbits. 3. Neurological conditions related to injury. 4. Neuropathy related to diabetes. 5. Pregnant or lactating. 6. Largely incapacitated or bedridden. 7. Enrolled in any other clinical study involving an investigational product or any other type of medical research judged not to be scientifically or medically compatible with this study or discontinued from any other clinical study involving an investigational product within 30 days of entry to the current study. 8. Patients who, in the investigator’s opinion, might not be suitable for the trial for safety reasons. |

**Supplementary Table 2.** Mean BRIEF-A, RBANS, MoCA, MIDAS, and HIT-6 scores at baseline, Week 8, and Week 16

| **Rating scale** | **Arm 1:  C1-INH → PBO* (N=18)** | **Arm 2:  PBO → C1-INH* (N=18)** |
| --- | --- | --- |
| BRIEF-A T-score  Baseline  Week 8  Week 16 | 66.0  61.8  62.4 | 66.1  62.9  60.4 |
| RBANS score  Baseline  Week 8  Week 16 | 97.1  98.9  94.4 | 97.3  99.8  99.4 |
| MoCA total score  Baseline  Week 8  Week 16 | 26.0  27.1  27.5 | 26.3  27.1  27.3 |
| MIDAS quantity score  Baseline  Week 8  Week 16 | 25.9  23.0  22.7 | 21.5  34.7  24.1 |
| MIDAS pain severity score  Baseline  Week 8  Week 16 | 5.4  5.2  4.6 | 4.1  4.5  3.6 |
| HIT-6 score  Baseline  Week 8  Week 16 | 56.6  54.6  54.7 | 54.1  51.7  48.8 |

*Patients were treated once a week, in Arm 1 with C1-INH (last dose at Week 7) and crossover to placebo (last dose at Week 15), and in Arm 2 with placebo (last dose at Week 7) and crossover to C1-INH (last dose at Week 15).

BRIEF-A, Behavior Rating Inventory of Executive Function-Adult; HIT, Headache Impact Scale; MIDAS, Migraine Disability Assessment; MoCA, Montreal Cognitive Assessment; RBANS, Repeatable Battery for the Assessment of Neuropsychological Status.
